# Pneumoperitoneum as a Complication of Mechanical Ventilation: A Case Report

**DOI:** 10.7759/cureus.41379

**Published:** 2023-07-04

**Authors:** Abbas A Mohamed, Mohammed Alharbi, Sarah Mohamed

**Affiliations:** 1 General and Laparoscopic Surgery, King Salman Specialist Hospital, Hail, SAU; 2 Medical College, Al Imam Mohammad Ibn Saud Islamic University, Riyadh, SAU; 3 General Surgery, University Hospital of Wales, Cardiff, GBR

**Keywords:** chest x-ray (cx-ray), conservative management.i, mechanical ventilation, idiopathic pneumoperitonium, non-surgical pneumopeitonium

## Abstract

Pneumoperitoneum is defined as the presence of free air in the abdominal cavity. The most common cause of pneumoperitoneum is intestinal perforation, which usually requires surgical intervention. Nonsurgical pneumoperitoneum (NPS) is defined as the presence of free air in the abdominal cavity without visceral perforation by an intrathoracic route, which commonly occurs in patients on mechanical ventilation in intensive care units. NSP, when properly diagnosed, can be successfully treated conservatively without surgery, and intensivists and surgeons should be aware of this entity associated with mechanical ventilation to avoid unnecessary surgical intervention.

## Introduction

Although the actual incidence of pneumoperitoneum associated with mechanical ventilation is unknown, current estimates range from rare to 7% of intubated intensive care unit (ICU) patients [1]. Unlike surgical pneumoperitoneum, which is associated with a perforation of a hollow organ, NSP associated with mechanical ventilation is clinically unremarkable and has no constitutional symptoms or signs of peritonitis. It usually presents as air under the diaphragm on an upright chest radiograph, without clinical signs of peritonitis. We report a patient who was mechanically ventilated and in whom air under the diaphragm on chest X-ray was an incidental finding. The patient was successfully treated without the need for surgery.

## Case presentation

**Figure 1:**
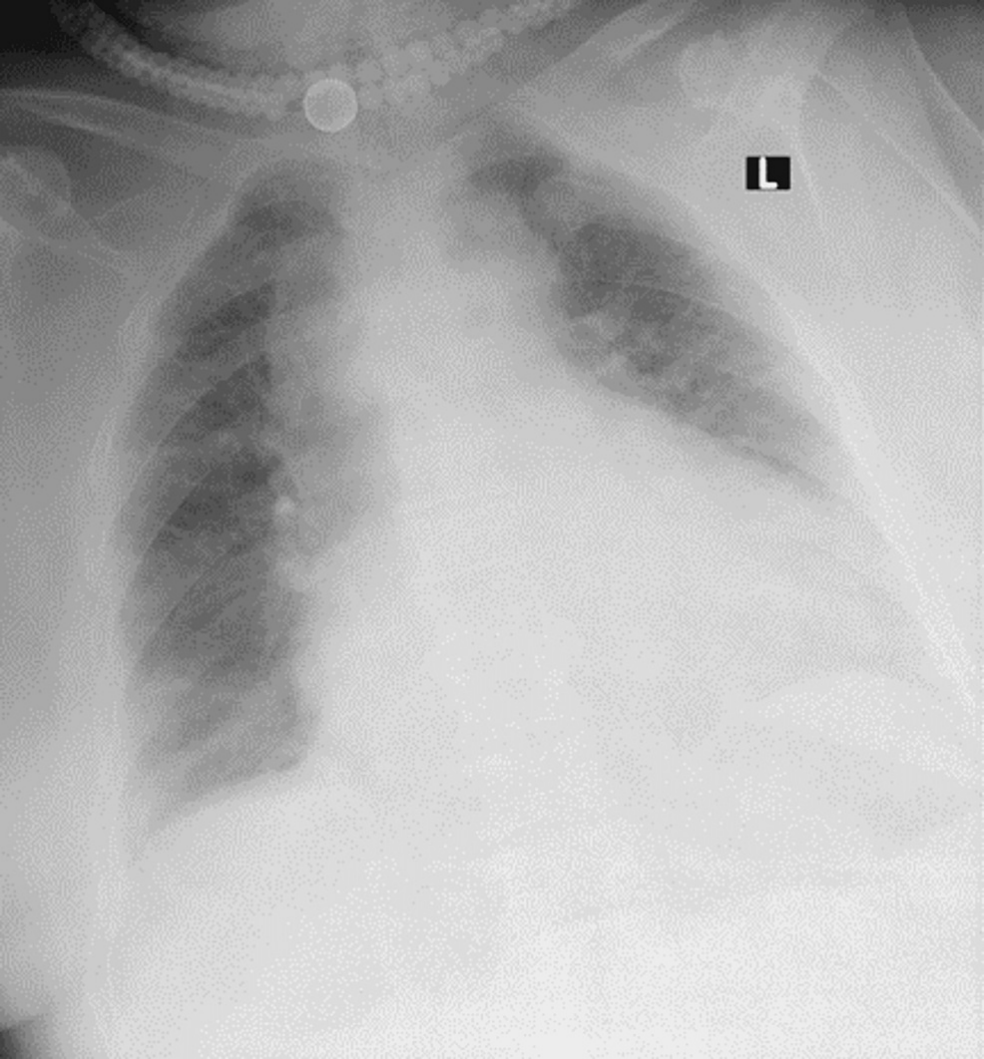
Plain chest radiography showing bilateral hilar and lung congestion with scattered ground-glass opacities, obliteration of both costophrenic angles, an enlarged cardiac shadow, and a widened upper mediastinum.

An 82-year-old woman with type 2 respiratory failure, obstructive sleep apnea, obesity hyperventilation syndrome, hypertension, recurrent stroke, and a paraumbilical hernia was admitted to our ICU with severe pneumonia. On admission and physical examination, the patient had tachycardia (pulse of 96 beats per minute), a blood pressure of 114/76 mmHg, fever (38.2 °C), tachypnoea (28 breaths per minute), and hypoxia (oxygen saturation of 88% on room air). A chest examination revealed bilateral basal crepitations on both sides of the chest. The abdomen was not distended and was soft but not tender. The hernia was irreducible, but there was no evidence of obstruction or strangulation. Chest radiography showed bilateral congestion of the hilar and lung with ground-glass opacities, obliteration of both costophrenic angles, an enlarged cardiac shadow, and a dilated upper mediastinum (Figure 1).

Laboratory investigations revealed a hemoglobin level of 9.3 g/L, a leucocyte count of 16.3 (109/L), and a platelet count of 165 (10^9^/L). Liver function, urea, creatinine, electrolytes, and serum amylase were within normal values. The coagulation profile was also within the normal range.

**Figure 2:**
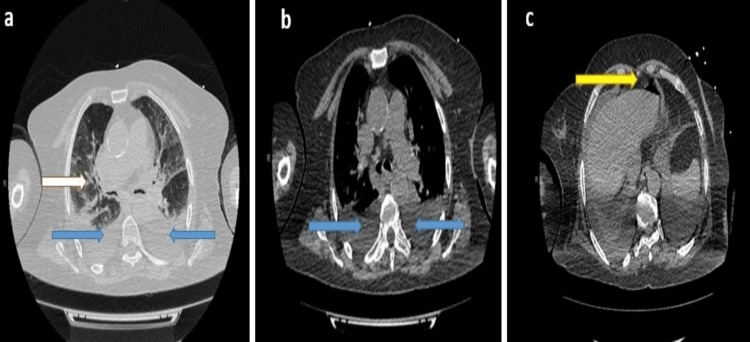
Computerized tomography chest scan: (a) and (b) mild cardiomegaly and pulmonary congestion, moderate-to-severe bilateral pleural effusions (blue arrows), and basal lung consolidation and collapse (white arrow); (c) pneumoperitoneum anterior to the liver (yellow arrow).

Continuous positive airway pressure therapy and intravenous antibiotic therapy were started, followed by daily chest radiographs. For further investigation, a computerized tomography (CT) scan of the chest was performed, which showed mild cardiomegaly and pulmonary congestion, moderate-to-severe bilateral pleural effusions, and bilateral basal lung consolidation and collapse (Figures 2a-2b). Upper abdominal scans showed pneumoperitoneum anterior to the liver (Figure 2c).

The radiologist suggested further investigation via a CT of the abdomen and pelvis with oral contrast. The surgical team was called in and confirmed that there was no evidence of peritonitis or hernia obstruction/strangulation.

**Figure 3:**
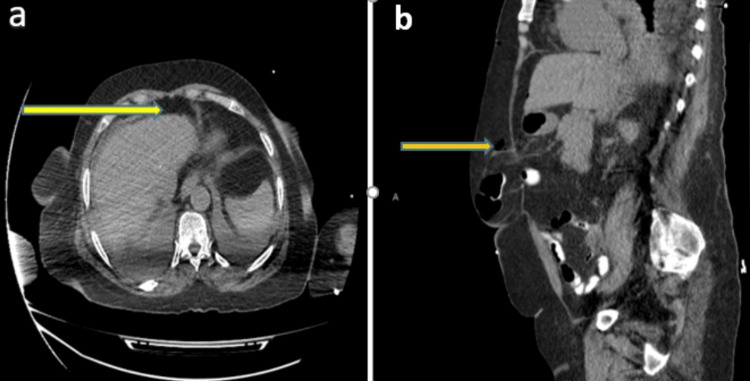
Abdominal CT scan with oral contrast: (a) air under the diaphragm (yellow arrows); (b) air within the subcutaneous tissue outside the hernia sac. Both figures show no leak of the contrast outside the bowel. CT, computerized tomography

The abdominal CT examination with oral contrast showed cardiomegaly with moderate bilateral pleural effusion and underlying consolidation/collapse. Pneumoperitoneum was seen anterior to the liver surface (Figures 3a), as was surgical emphysema directly overlying the paraumbilical hernia sac without significant contrast leakage at the time of examination (Figures 3b).

The patient was given oral fluids and gradually progressed to normal nutrition without abdominal pain or signs of peritonitis.

## Discussion

Pneumoperitoneum is a radiological term indicating an abnormal accumulation of air in the peritoneal cavity. In patients who have not had a recent laparotomy or laparoscopy, pneumoperitoneum indicates the rupture of an intra-abdominal viscus in approximately 90% of cases [2]. The remaining 10% of cases are due to physiological processes that do not require surgical intervention and are referred to as nonsurgical pneumoperitoneum (NSP).

Pneumoperitoneum not associated with visceral perforation has taken various names in the past, such as aseptic spontaneous pneumoperitoneum, idiopathic pneumoperitoneum, spontaneous asymptomatic pneumoperitoneum, and NSP [3].

The most common abdominal ethology of NSP is retained postoperative air (25%-60%), peritoneal dialysis catheter placement (10%-34%), and after gastrointestinal endoscopic procedures (0.3%-25%). The most common thoracic causes include mechanical ventilation, cardiopulmonary resuscitation, and pneumothorax [1].

The exact mechanism of NSP in ventilated patients is unclear. Donahoe et al. suggested that NSP may occur secondary to a pulmonary air leak that spreads from the mediastinum through perivascular spaces into the retroperitoneum and then ruptures into the peritoneum [4]. This suggestion was supported by Turner and Fry, who reported that autopsy investigations in two mechanically ventilated patients who developed pneumoperitoneum revealed extraperitoneal subdiaphragmatic emphysema with secondary rupture into the free peritoneal cavity [5]. In contrast with pneumoperitoneum associated with visceral perforation, NSP usually presents as air under the diaphragm on an upright chest radiograph, without clinical signs of peritonitis.

An erect chest X-ray is the most sensitive plain radiograph to detect free intraperitoneal gas in an emergency setting [6]. The presence of free air in the peritoneal cavity in an abdominal radiograph is associated with various relevant described signs depending on the amount of air and the place of its collection. These signs include the Rigler sign (i.e., gas on both sides of the intestinal wall), the falciform ligament sign (i.e., gas at the falciform ligament), the football sign (i.e., gas outlining the peritoneal cavity), and the inverted *V* sign (i.e., the presence of air in the medial umbilical fold) [7].

NSP poses significant management problems for surgeons, especially when sedated and mechanically ventilated patients in the ICU have no signs of peritonitis or when it is difficult to detect. The diagnosis is usually made after a negative laparotomy result but can be successfully observed without surgery if the preoperative diagnosis is correct. ICU physicians and surgeons must be aware of this entity of NSP associated with mechanical ventilation to avoid adverse laparotomies and associated morbidity and mortality in this patient group.

Okamoto et al. conducted a retrospective midline search of all published reports of NSP during mechanical ventilation between January 1990 and December 2013, which yielded a total number of 115 cases in adults [8]. All cases were associated with either pneumothorax or pneumomediastinum, or both, and usually, these complications preceded the development of pneumoperitoneum. Surprisingly, of the 115 reported cases, 57 underwent surgical treatment without evidence of a perforated bowel.

Mularski et al. retrospectively studied cases of pneumoperitoneum from non-surgical causes at the two teaching hospitals of a university hospital between January 1990 and December 1995 [1]. During this period, eight [PW3] patients (six men and two women; mean age = 61 years) were identified to have non-surgical causes of pneumoperitoneum. Two patients (25%) underwent negative laparotomy, and the other six (75%) were successfully treated non-operatively and discharged from the hospital.

Van Gelder et al. reported seven cases of NSP admitted over three years to Grady Memorial Hospital, Atlanta, Georgia. Six patients with pneumoperitoneum underwent exploratory laparotomy when clinical examination suggested an acute abdomen, but none were found to have intra-abdominal pathology. A seventh patient who was ventilated was treated conservatively after a diagnostic peritoneal lavage was performed, which was negative [9]. Although most reported cases of NSP are usually preceded by either pneumothorax or pneumomediastinum, our case was not associated with either for unknown reasons.

## Conclusions

The most common abdominal cause of NSP is retained postoperative air, peritoneal dialysis catheter placement, and after gastrointestinal endoscopic procedures, whereas the most common thoracic causes include mechanical ventilation, cardiopulmonary resuscitation, and pneumothorax. NSP is a rare complication of mechanical ventilation that usually presents as air under the diaphragm on an upright chest radiograph without evidence of peritonitis. The diagnosis of NSP associated with mechanical ventilation can be difficult for surgeons, especially in sedated patients. A CT scan with oral contrast can help with the diagnosis by proving that there is no leak from the bowel.

Surgeons and intensivists must be aware of NSP associated with mechanical ventilation to avoid unnecessary surgical intervention. Conservative treatment is appropriate in the absence of symptoms or signs of peritonitis.
